# Macroporous PES Composite Membranes Built on an sPES-Containing Asymmetric Platform for Improved Fermentation-Broth Filtration

**DOI:** 10.3390/membranes16100318

**Published:** 2026-09-25

**Authors:** Soyoung Moon, Jaehyun Yoo, Yongmin Shin, Sungryul Park

**Affiliations:** UMTR Co., Ltd., Ochang, Cheongju 28116, Republic of Korea; sy@umtrbio.com (S.M.); yjh@umtrbio.com (J.Y.); crecredit@umtrbio.com (Y.S.)

**Keywords:** polyethersulfone, sulfonated PES, composite membrane, asymmetric microfiltration, fermentation broth, phase separation, interlayer contact

## Abstract

High clean-water throughput does not necessarily translate into rapid fermentation-broth filtration. We hypothesized that a macroporous upstream layer combined with an asymmetric polyethersulfone (PES) membrane could unite rapid filtration at low broth concentration with improved performance under a greater fouling challenge. Development progressed from symmetric and asymmetric manufacturing platforms through layered proof-of-concept tests to a PES composite. Preliminary asymmetric-PES/nitrocellulose assemblies increased average broth flux 4.81-fold at 100-fold dilution and 3.74-fold at 50-fold dilution relative to their respective bare-base controls. A separately cast PES upstream layer was then developed using mixed polyethylene-glycol additives and vapor/non-solvent-induced phase separation. The selected 40 µm layer had an open particulate morphology and a clean-water flux of 147,000 L m^−2^ h^−1^. Its composite filtered 100 mL of 50-fold diluted *Escherichia coli* broth in 52.35 s versus 185 s for an asymmetric-base reference. Subsequent upstream incorporation of 0.5 wt% acid-form sulfonated PES improved composite broth flux 1.77–2.25-fold despite lower isolated-layer water flux; both tested coupons exceeded two commercial references in the same session. The final PES and PES/sPES-H composites were compared at 50-fold dilution only. These results support the architectural hypothesis and establish a third-generation PES composite through coordinated development of upstream morphology, composition and interlayer contact.

## 1. Introduction

Polyethersulfone (PES) microfiltration membranes can be manufactured with a broad range of porous structures by controlling casting-solution composition and phase separation. Experimental studies of hydrophilic PES microfiltration have demonstrated the importance of the additive package and vapor exposure in determining membrane morphology and filtration behavior [1,2,3,4,5]. Macromolecular additives also affect the surface properties, permeability and stability of phase-inversion PES membranes [6,7,8,9,10,11,12]. These variables provide a practical route for developing membranes that combine a controlled nominal grade with high water throughput.

The challenge changes when the feed is a fermentation broth [13,14,15]. A membrane that passes water rapidly may slow markedly when exposed to cells, debris and other broth constituents. Pore blockage and deposit formation provide a physical basis for this difference: the resistance during filtration evolves with the feed and the material already deposited on the membrane. The combined pore-blockage and cake-filtration model of Ho and Zydney [16] and the related blocking-law treatments [17,18,19] describe this coupling for protein and cell microfiltration. In the present development, the relevant engineering question was therefore whether a membrane could retain the fast response observed with diluted broth while avoiding the large performance penalty encountered with a more concentrated broth.

This work should be read as the first experimental stage of a longer development hypothesis. High-cell-density bacterial fermentation broths, such as *E. coli* cultures, present a fouling load that a single microfiltration sheet cannot process economically in normal-flow (dead-end) mode, and the authors’ working hypothesis is that a membrane suited to such feeds will require a deliberately engineered multilayer architecture of two, three or more layers, each assigned a different depth-retention and flow function, rather than a single sheet with a finer or coarser rating. Tangential-flow filtration remains the established industrial method for clarifying whole broth [13,14,20], and it is not displaced by this hypothesis; the motivation for pursuing a normal-flow route is that a membrane that removes as much cell mass and debris as possible in a single pass can shorten or simplify the clarification train and can be used directly in the single-use, vacuum- or pressure-driven devices supplied by the commercial references of this study. Recent sterile-filtration studies of complex biological feeds show that the choice and structure of the upstream (prefilter) layer, rather than the rating of the final membrane, frequently determines the achievable throughput [21,22,23]. The present article reports the first step of that sequence: it establishes whether an independently designed upstream layer on the asymmetric base changes broth filterability in the intended direction. The broth was therefore diluted by fixed factors (50-fold and 100-fold) to deliver a reproducible and measurable fouling load to a fixed area within a 100 mL charge, so that membranes and assemblies could be ranked on the same prepared feed. These dilutions are test-severity levels chosen to expose the architectural effect at an early stage, not proposed process conditions; a 50-fold dilution of a production fermenter is evidently impractical, and the ability to treat undiluted or lightly diluted broth is the target of the next generation of the architecture, as discussed in Section 3.10.

The manufacturing platform was developed in three generations. First-generation symmetric PES membranes (SY) established the casting and pilot-production basis. Second-generation asymmetric membranes (ASY) extended that platform into a higher-throughput manufacturing regime. Both generations included PES-only and sPES-containing formulations; sulfonated PES (sPES) incorporation itself is consequently not the generation boundary. The third generation adds an independently designed macroporous upstream region to an sPES-containing asymmetric base (Figure 1). This architecture permits adjustment of the feed-contacting structure while retaining the established base-manufacturing route.

The original hypothesis was that this composite architecture could combine the strengths sought at both low and higher broth concentrations. In early screening, an in-house ASY sample exceeded the Sartorius reference at 100-fold dilution, whereas a stronger broth challenge exposed the limitations of a single asymmetric sheet. The commercial references also behaved differently: Sartolab 500 (Sartorius) was faster than Steritop (Millipore) at 100-fold dilution, while Steritop was faster at 50-fold dilution in the corresponding screening blocks. These observations defined a practical target: a membrane with rapid dilute-broth filtration and an improved response when more broth material was presented to the membrane. Commercial membranes were used to locate the in-house performance within each comparison, with the same test feed used for the membranes compared in that session.

There is an established rationale for introducing an upstream porous region. Recent work on sterilizing membranes and prefilters showed that an integrated prefilter can improve filtration performance for an mRNA–lipid-nanoparticle feed [24,25,26,27]. Although that feed differs from bacterial broth, it demonstrates that nominal membrane rating alone does not determine application performance [28,29]. Combined vapor-induced and non-solvent-induced phase separation (VIPS/NIPS) has also been used to prepare isotropic macroporous PES components for composite membranes [30]. The present work applies this structural flexibility to a feed-facing layer above an asymmetric microfiltration base rather than to a support below a newly polymerized selective film.

The use of sPES provides a further compositional variable. PES/sPES blends have been investigated at both membrane and module scales for their mechanical and fouling behavior [31,32,33,34,35,36,37], while recent PES microfiltration work has emphasized the role of surface modification in protein-containing feeds [38,39]. The sPES additive used in the base formulation and its effect on the bubble point–permeability balance of roll-to-roll PES microfiltration membranes are described in a companion study [40]. Here, sPES was already present in the selected ASY base. Acid-form sPES was subsequently introduced into the upstream layer as an additional refinement within the composite architecture. The central contribution is the development and experimental evaluation of that architecture, with the later sPES-H formulation treated as part of the same development sequence.

We report the manufacturing background, the preliminary layered tests at two broth dilutions, the development of a thin macroporous PES layer, and its assembly and comparison with the ASY base and commercial references. The experimental argument follows the original hypothesis: establish the concentration-dependent need, test the benefit of an upstream layer, and implement the concept as a PES composite using controlled formulation, phase separation and contact between the completed membranes.

## 2. Materials and Methods

### 2.1. Materials, Datasets and Membrane Designations

Polyethersulfone (PES; Ultrason E 6020 P, BASF, Yeosu, Republic of Korea) and in-house sPES (synthesized as described in the companion study [40]) were used as membrane-forming polymers. Casting formulations contained polyvinylpyrrolidone (PVP K-30; Samchun Chemical, Pyeongtaek, Republic of Korea), polyethylene glycol (PEG 200, PEG 400 or PEG 2000; Samchun Chemical), lithium chloride (Samchun Chemical) or calcium chloride (Junsei Chemical, Tokyo, Japan), and dimethyl sulfoxide (DMSO, >99.5%; Samchun Chemical); ethanol and butanol (99.5%; Samchun Chemical) were used in the selected base formulation. All compositions are expressed as mass percentages of the complete casting dope. The principal formulations are listed in Table 1. Acid-form sPES used in upstream dope 260831-2 is designated sPES-H. The counterion form of the sPES in the historical base-production record is not assigned as H-form.

The historical SY/ASY dataset is the released manufacturing dataset of the companion manufacturing-data study [41]. It contains one consolidated record per casting sample, including formulation, process and performance variables. The standard analysis cohorts comprise 114 SY records from 18 March to 11 November 2024 and 332 ASY records from 16 September 2025 to 8 July 2026. Exact bubble-point and water-throughput measurements are jointly available for 69 and 272 samples, respectively. Missing values were retained; measurements without an observed bubble-through endpoint were excluded from calculations requiring an exact bubble point. Table 2 summarizes the cohorts. Their role here is to establish the manufacturing platform; the composite fabrication and broth comparisons constitute the subsequent experimental development.

ASY-base denotes the sPES-containing asymmetric component. Composite-PES denotes an ASY base combined with a separately formed PES upstream layer, while Composite-sPES-H denotes the corresponding assembly with a PES/sPES-H upstream layer. Original casting identifiers are retained. Initial proof-of-concept assemblies used an in-house nitrocellulose membrane, NC 5K11-5, on an ASY base; these are identified separately as ASY + NC. NC is not a component of the final PES composite. The commercial references were the membranes of two single-use vacuum bottle-top filter units: Sartolab BT 500 (Sartorius, Göttingen, Germany; cat. no. 180E14---------E; 0.22 µm asymmetric polyethersulfone membrane, effective filtration area 69 cm^2^ according to the manufacturer) and Steritop 500 mL (Merck Millipore, Burlington, MA, USA; cat. no. S2GPT05RE; 0.22 µm Millipore Express PLUS polyethersulfone membrane, 40 cm^2^). Both are sterilizing-grade PES membranes; “Sartolab 500” and “Steritop” in the tables and figures denote the membranes of these units, not the housings. The February–July bottle-top tests used the membranes in their original housings; for the 2 September comparison, 47 mm coupons were cut from the same unit membranes and tested in the common holder. Nominal pore ratings are the manufacturers’ designations, and all reported reference performance was measured in-house.

### 2.2. Pilot Manufacture of the Asymmetric Base

The ASY base was manufactured on a continuous roll-to-roll slot-die line with a PET release liner, multistage humidification, aqueous coagulation and a three-zone dryer. The selected dope, 260625-1, contained 11.5 wt% PES, 0.5 wt% sPES, 30 wt% PEG 200, 0.5 wt% PVP K-30, 1 wt% CaCl_2_, 5 wt% ethanol, 5 wt% butanol and 46.5 wt% DMSO. Its recorded viscosity was 1146 cP. This composition is also retained in the released dataset as dope lot A-L079.

Base 6G01-4 was produced at a web speed of 0.6 m min^−1^, with four humidification stages, a NIPS bath set to 50 °C (recorded bath temperature 52 °C) and three-zone dryer settings of 50, 50 and 60 °C. The pilot slot-die gap was 300 µm and the recorded membrane width was 502 mm. The remaining dope was used for the subsequent 50 m run 6G01-6 under the same recorded nominal settings. These bases share a dope and process history but retain separate sample identifiers. The upstream layer was placed on the larger-pore face of the base, toward the incoming broth.

### 2.3. Preparation of the Macroporous Upstream Layer

The early laboratory dope, 260710-1, contained 8 wt% PES, 0.5 wt% LiCl, 6 wt% PVP K-30, 16 wt% PEG 400 and 69.5 wt% DMSO, with a viscosity of 738.8 cP. Films were cast at nominal wet thicknesses of 100 or 150 µm, exposed to humid air for 10 or 20 min, coagulated in water at 40 or 60 °C for 10 min, and dried at 50 °C for 20 min. These conditions produced dry layers of 20–30 µm. The casting matrix and isolated-layer water measurements are reported in Table 3.

The revised formulation, 260728-3, retained 8 wt% PES and 0.5 wt% LiCl, reduced PVP K-30 to 1 wt%, increased PEG 400 to 20 wt%, and introduced 5 wt% PEG 2000, with 65.5 wt% DMSO. Its viscosity was 276 cP. Carrier trials led to selection of polypropylene (PP): an RX600 carrier did not permit release, while a 150 µm wet film on PP folded during coagulation. The selected route used a 250 µm wet casting thickness on PP, 15 min vapor exposure and 20 min aqueous coagulation. The temperature pair 260728-3-5 and 260728-3-6 used 30 and 60 °C baths, respectively, with the other recorded settings held constant. Both were dried at 50 °C for 20 min. PP served as a temporary casting carrier and was removed from the completed membrane.

Later dopes 260811-1 and 260825-1 reproduced the revised PES formulation family. Dope 260825-2 changed PEG 400/PEG 2000 to 15/6 wt%; 260825-3 reduced PES to 7.5 wt%. The subsequent 260831-2 formulation replaced 0.5 wt% of the PES with sPES-H, giving 7.5 wt% PES and 0.5 wt% sPES-H while retaining the principal additive package. Its recorded viscosity was 301 cP. Extending coagulation from 20 to 40 min raised the composite broth flux from 971 to 1130 L m^−2^ h^−1^ (52.35 to 45.02 s for 100 mL) in the 31 July block, and 40 min coagulation was adopted for the later layers. Casting identifiers in the September comparison distinguish individual layer specimens within these dope families.

### 2.4. Layered Proof of Concept and Composite Assembly

The preliminary ASY + NC tests combined base 6C13-2 with NC 5K11-5, with or without an additional nominal 50 µm mesh. Wet-contact and simply stacked configurations were recorded in the 100-fold-dilution block. The 50-fold block included the bare base, ASY + NC, and ASY + NC + mesh. These configurations tested the architectural concept before the independently cast PES upstream layer was established. Table 4 presents the original configurations and elapsed times.

For the final composite route, the PES upstream layer and ASY base were completed separately and assembled with the upstream layer facing the feed. In the later wet-consolidation procedure, the membranes were stacked while wet, and 50 mL of deionized water was passed through the pair at a recorded nominal pressure of 1 bar to establish contact. Loose stacks were included for comparison. Wet consolidation describes the assembly procedure and does not imply a permanent adhesive or a measured bond strength.

Attempts to establish contact during coagulation included pretreatment of the base with 20 wt% aqueous glycerol and with 50 wt% DMSO/water for 30 s, 1 min or 5 min. The records followed conformity and separation during immersion and drying. These trials informed the eventual use of wet consolidation after both membranes had formed. Contact comparisons therefore assess the practical assembly condition, which can include differences in wetting and interlayer air as well as conformity.

### 2.5. Bubble Point, Water Flow and Electron Microscopy

Bubble point was measured using a Palltronic Flowstar IV instrument (Pall Corporation, Port Washington, NY, USA) on deionized-water-wetted coupons pressurized with nitrogen at 22 °C, following the laboratory procedure based on ASTM F316 [42]. The endpoint setting was 7000 mbar; tests without observed bubble-through were recorded as censored. The nominal 0.2 µm designation identifies the ASY base grade. Bubble point and throughput were retained as separate measurements. Bacterial retention and protein adsorption of the 0.2 µm base grade were determined by an external laboratory (Membrane Laboratory, Department of Biological Engineering, Inha University, Incheon, Republic of Korea) on 13.8 cm^2^ coupons at 1 bar. The bacterial challenge procedure consisted of a water-flux measurement, buffer flushing, filtration of 200 mL of a ~10^7^ CFU mL^−1^ suspension of *Brevundimonas diminuta* in phosphate-buffered saline (pH 7.4), plate counting of the permeate and calculation of the challenge level per unit membrane area. Protein adsorption was measured on separate coupons by filtering 250 mL of 1 g L^−1^ bovine serum albumin in the same buffer and quantifying the adsorbed amount. A Millipore Express PLUS 0.22 µm PES membrane was tested in parallel as the reference in every series.

Clean-water throughput was determined gravimetrically on 47 mm coupons. The exposed diameter inside the gasket was 40.4 mm, corresponding to a rounded effective area of 12.8 cm^2^. At a water density of 1 g mL^−1^, a flow of 1 g min^−1^ corresponds to 46.875 L m^−2^ h^−1^ on that area. Water fluxes in this article use that conversion consistently; source flow rates are retained in the tables. Values are fluxes rather than pressure-normalized permeances because a measured driving pressure is not available for every historical entry.

Scanning electron micrographs were used to compare the surfaces and cross-sections of early and revised upstream layers, an ASY base and the commercial references. In-house images were acquired at 10 kV, with nominal surface magnification of 5000× and cross-section magnifications of 400–2000×, as indicated by the original image metadata. Images from a second instrument were retained for Sartolab 500. Original scale bars were preserved. Each panel is identified by its specimen; separate component cross-sections show the constituent morphologies, not the assembled interlayer boundary. Surface porosity and pore-opening size distributions were quantified on the ×5000 surface fields used in the morphology comparison of Section 3.5 (early, revised and later upstream layers, the ASY base and the two commercial references) with an in-house image-analysis tool (SEM/Pore Analysis v4.0; UMTR Co., Ltd., Cheongju, Republic of Korea), which follows an ImageJ-type workflow. Each field was calibrated from the recorded horizontal field width (25.62 µm over 1536 px, 0.01668 µm px^−1^; 0.05 µm px^−1^ for the Sartolab field from the second instrument), the 88 px instrument footer was cropped, and the image was contrast-stretched (1–99th percentile) and smoothed (Gaussian σ = 0.6 px). Pore openings were segmented by seed-and-grow (hysteresis) thresholding of the dark phase, with a closing of 2 px and a minimum object diameter of 0.08 µm; the seed/grow gray-level thresholds were adjusted per field (26–53/60–110 on an 8-bit scale) to follow the depth contrast of each morphology, and manual corrections were applied only to the early layer (27 operations) and the Sartolab field (38 operations) where particulate debris or surface texture overlapped the openings. Surface porosity is the two-dimensional projected area fraction of pore openings within the analysis region; it is not a bulk porosity. Objects touching the field border were included in the porosity but excluded from size statistics. Each object is characterized by its equivalent circular diameter, D_eq = 2(A/π)^1/2^, and its largest inscribed diameter; number-weighted medians, means, 90th percentiles (D90) and maxima are reported, together with the area-weighted mean diameter. All measurements, masks and settings are archived per field.

### 2.6. Broth Filtration and Comparison Basis

*Escherichia coli* fermentation broth supplied by UNIST was diluted with deionized water. The experimental description identifies the strain as K-12 MG1655. The historical screening and initial layered tests used 100-fold and 50-fold dilutions. These are operationally designated the lower- and higher-concentration conditions; they are relative dilutions of broth, not universal solid-concentration thresholds. The July and September PES-composite comparisons used 50-fold dilution. Broth lots differed between development sessions, including material received in May and a later lot received after the supplier’s ion-exchange processing in August. Relative membrane performance was assessed using the same prepared broth for the membranes compared within each experimental block.

A 100 mL charge was filtered in dead-end mode, and the elapsed time to completion was recorded. Filtration was vacuum-driven: the recorded setting was a nominal 1 bar, and the 2 September session used a house vacuum of approximately −1 bar (gauge) on the filtrate side. Fluxes are calculated from volume, area and time only and are not pressure-normalized. February–July bottle-top tests used effective areas of 70.84 cm^2^ for in-house membranes, 69 cm^2^ for Sartolab 500 and 40 cm^2^ for Steritop. The 2 September comparison was performed on a single day with the same prepared broth, the same 47 mm holder (12.8 cm^2^ effective area) and the same vacuum setting for all membranes, including commercial coupons removed from their housings. Device-specific bottle-top results describe performance in those test configurations; the September comparison provides the common-holder comparison.

The primary performance measure was average flux over the fixed charge, $\bar{J}$ = V/(At), where V is filtrate volume, A is effective area and t is elapsed time. With V = 100 mL, A in cm^2^ and t in seconds, $\bar{J}$ = 3,600,000/(At) L m^−2^ h^−1^. Thus, the conversion factors are 50,818.75/t for the in-house bottle-top and 281,250/t for the 47 mm holder. The corresponding volume loads are 14.12 and 78.13 L m^−2^. Times and fluxes from these different configurations were not pooled. Derived fluxes are reported to three significant figures, which reflects the precision of the timed 100 mL endpoints and the effective areas; recorded times, masses and flow rates are given as recorded, and ratios were calculated from the unrounded values.

For comparisons on a common area and volume, the flux ratio is the inverse elapsed-time ratio, ${\bar{J}}_{\mathrm{test}}$/${\bar{J}}_{\mathrm{reference}}$ = t_reference_/t_test_. For different bottle-top areas, the stated areas were included explicitly. The absolute mass and composition of broth solids were not used to calculate these ratios. Consequently, relative filtration performance can be quantified within an experimental block without assigning a fully resolved solids concentration to the feed. Commercial membranes serve as measured references for that block.

### 2.7. Data Processing

Historical cohort summaries were calculated from the canonical CSV files. Broth endpoints were transcribed from the original dilution workbook, the composite-development presentation and the authors’ specimen-level comparison tables. Repeated commercial entries are reported individually, and repeated appearances of the same entry in summary columns were not counted as additional experiments. The early July worksheet combines a 2 July header with later casting identifiers; those rows are therefore identified as an earlier development block, while the explicit 31 July block is reported separately.

Ratios describe recorded screening outcomes. The two consolidated sPES-H coupons are separate specimens from one dope, not independent manufacturing-batch replicates. The isolated-layer water range for dope family 0831-2 is a reported range across specimens and is not a confidence interval or a matched water measurement for each broth coupon. Single endpoints are shown without inferential significance tests. Mechanistic explanations are discussed as interpretations of the observed structure, formulation and assembly responses.

## 3. Results and Discussion

### 3.1. SY-to-ASY Development Provides a High-Throughput Base

The historical cohorts establish a manufacturing progression that preceded the composite work. SY includes 73 PES-only and 41 sPES-containing records; ASY includes 96 and 236, respectively (Table 2). The structure and processing route, rather than the presence of sPES alone, distinguish these two generations. In the joint bubble-point/throughput subsets, median water flow increased from 290.33 g min^−1^ for SY to 365.80 g min^−1^ for ASY, corresponding to approximately 13,600 and 17,100 L m^−2^ h^−1^. Median bubble points were 3733 and 3550 mbar, while median thicknesses across available observations were similar, at 157 and 156 µm.

The approximately 26% water-throughput contrast illustrates the higher-throughput region occupied by the ASY manufacturing platform (Figure 2a). The cohorts span different production periods and formulations, so this comparison is a manufacturing outcome rather than an isolated effect of asymmetry or sPES. It establishes that the third-generation work began with a developed base, not an unoptimized reference film.

The selected 6G01-4 base further improved on the historical ASY median. Its recorded cross-web bubble points were 4100, 4150 and 3700 mbar, with water flows of 511, 611.8 and 595 g min^−1^, equivalent to approximately 24,000, 28,700 and 27,900 L m^−2^ h^−1^. The related 6G01-6 run used the remaining dope and the same nominal process. These records connect the manufacturing platform to the base component used in the composite experiments.

### 3.2. Dilution-Dependent Screening Defines the Architectural Hypothesis

The early broth comparisons show why high throughput at low concentration was an incomplete design criterion. At 100-fold dilution, ASY sample 5J20-1 filtered 100 mL in 4.86 s, corresponding to 10,500 L m^−2^ h^−1^ on the in-house bottle-top area. In that screening block, the Sartorius entries were 7680 and 8980 L m^−2^ h^−1^, and the Millipore entries were 5650 and 6470 L m^−2^ h^−1^ (Table 4; Figure 3a). The in-house ASY sample therefore exceeded both references under the recorded lower-concentration condition.

At 50-fold dilution, the corresponding historical ASY entry required 138 s and gave 368 L m^−2^ h^−1^. Sartorius gave 202–237 L m^−2^ h^−1^, whereas Millipore gave 2730–2920 L m^−2^ h^−1^ (Figure 3b). Thus, the ASY membrane retained an advantage over Sartorius but fell well below Millipore in the stronger-broth screening block. The February blocks were measured on different dates, so the change is not used to fit a concentration-response law. The relevant observation is the performance ranking within each block: the low-concentration throughput advantage did not by itself resolve the higher-concentration challenge.

This distinction led to the original composite hypothesis. An open upstream region could alter how broth interacts with the membrane assembly while the asymmetric base supplies the established microfiltration structure. The engineering objective was to maintain rapid filtration in dilute feeds and improve filtration when the feed imposed a larger fouling challenge. The commercial references defined useful performance levels during this development; their catalog specifications were not substituted for measured controls.

### 3.3. Initial Layered Tests Support the Hypothesis at Both Dilutions

The initial ASY + NC experiments directly tested whether an additional upstream membrane could improve the response at both operational concentration levels. In the 100-fold-dilution block, bare base 6C13-2 required 66.52 s for 100 mL, while wet-contact assembly with NC 5K11-5 required 13.84 s. Average flux increased from 764 to 3670 L m^−2^ h^−1^, a 4.81-fold improvement. A simply stacked ASY + NC entry required 19.31 s, and the assembly with mesh required 19.43 s (Table 4; Figure 3c).

The March bare-base value (764 L m^−2^ h^−1^ for 6C13-2 at 100-fold dilution) is much lower than the February value for ASY 5J20-1 under the same nominal dilution (10,500 L m^−2^ h^−1^; Figure 3a). The two entries are not the same membrane: 5J20-1 and 6C13-2 are different casting samples, and 6C13-2 was cast from a different formulation (12.0 wt% PES/1.4 wt% sPES, dope viscosity 2082 cP; Table 5) with a different humidification setting from the later 11.5/0.5 wt% base family. They were tested on different dates from the same broth delivery, which was diluted freshly for each block, so that the broth had been stored for about six additional weeks before the March block; the dilution factor fixes only the volume ratio, not the cell and debris load, which changes with storage and differs between fermentation batches. For these reasons the February and March values are treated as separate blocks and are compared only within a block, as stated in Section 2.6; the difference between blocks is not interpreted as a membrane property. This block-to-block variation is itself part of the motivation for reporting same-session, same-feed comparisons throughout the paper.

In the 50-fold-dilution block, the bare base required 224.46 s, compared with 60.02 s for ASY + NC. Flux increased from 226 to 847 L m^−2^ h^−1^, a 3.74-fold improvement. The ASY + NC + mesh configuration reached 22.61 s and 2250 L m^−2^ h^−1^, approximately 9.93 times the bare-base value (Figure 3d). The latter stack had a total recorded thickness of 412 µm, compared with 155 µm for the bare base and 335 µm for ASY + NC.

These paired comparisons support the central architectural hypothesis at both tested dilutions: the benefit of an upstream layer was not confined to only the more dilute or the stronger-broth condition. They also show that contact and the arrangement of the layers matter. The NC-based assemblies are an early proof of concept, distinct from the later PES composite. Their role was to establish the value of a layered architecture and motivate an upstream PES component whose thickness, morphology and composition could be developed independently.

### 3.4. Base Selection Requires Broth Performance as Well as Water Throughput

The April comparison sharpened the selection criterion for the asymmetric base (Table 5). Samples 6C13-2 and 6D14-1 had the same reported bubble point, 3650 mbar, and nearly equal water fluxes, 18,100 and 18,400 L m^−2^ h^−1^. Their 50-fold-broth fluxes differed substantially, at 226 and 560 L m^−2^ h^−1^, a ratio of 2.47. At 100-fold dilution the recorded values were 847 and 953 L m^−2^ h^−1^, a ratio of 1.12 (Figure 2b).

The samples differed in polymer composition, dope viscosity and active humidification stages. The combined formulation/process change, rather than any single ingredient, is associated with the better broth response. More importantly for the present design, equal bubble point and almost equal water flux did not predict equal broth filterability. This observation justified retaining broth filtration as the deciding application test while continuing to use water measurements and bubble point for manufacturing characterization.

### 3.5. Development of a Thin, Open PES Upstream Layer

Transfer of the layered concept to a PES upstream film was not achieved by simple stacking. In the earlier July development block, 6G01-4 alone required 153 s for 100 mL of 50-fold broth. The addition of a separately cast PES layer increased total thickness from 153 to 223 µm and increased filtration time to 351.2 s (Table 6). Early 260710-1 layers also slowed filtration: the V10 and V20 assemblies required 347 s and more than 600 s, respectively. The added porous film could therefore become a net resistance when its structure or assembly was unsuitable.

Reducing thickness alone did not solve this problem. The early 260710-1 formulation produced a 20 µm layer at 100 µm wet casting thickness, but wrinkling occurred after drying. At 150 µm wet casting thickness, the films were approximately 30 µm thick. Their isolated-layer water flow responded strongly to processing: 87.6 g min^−1^ at 10 min VIPS and 40 °C NIPS, 334.8 g min^−1^ when the bath was raised to 60 °C, and 808 g min^−1^ when vapor exposure was extended to 20 min (Table 3). These changes established sensitivity to phase separation but did not guarantee a beneficial composite.

The early surface and cross-section images show a relatively continuous surface perforated by discrete openings above a porous interior (Figure 4a,b). The development record described openings of approximately 1–3 µm, below the intended 10–15 µm design target. This target was a formulation objective, not a measured pore-size distribution for the final membrane. The distinction between an internally porous film and an adequately open external surface was central to the subsequent revision.

The revised 260728-3 dope changed the additive package while retaining 8 wt% PES. Reducing PVP K-30 from 6 to 1 wt%, increasing PEG 400 from 16 to 20 wt% and adding 5 wt% PEG 2000 lowered the recorded viscosity from 738.8 to 276 cP. These constituents changed together, so the outcome is attributed to the formulation package. The sensitivity is consistent with experimental literature on macromolecular additives and combined VIPS/NIPS processing of PES [1,2,6,30]. Carrier release and wet-film handling were addressed at the same time by selecting PP and a 250 µm wet casting thickness.

The two PEG grades are assigned complementary roles in this interpretation. PEG is a hydrophilic, water-miscible pore-forming additive whose molecular weight sets both its contribution to dope viscosity and the rate at which it is extracted during phase separation [7]. PEG 400, present at 20 wt%, is a low-molecular-weight component that lowers the dope viscosity, increases water uptake of the film during humid-air exposure and reduces the thermodynamic stability of the PES/DMSO solution, so that liquid–liquid demixing begins early in the VIPS stage. Under these conditions, demixing proceeds by nucleation and growth of the polymer-lean phase, which produces the interconnected particulate (nodular) polymer network seen in Figure 4c–f rather than a dense skin [10,11,43]. PEG 2000, at 5 wt%, is extracted more slowly and is expected to remain in the polymer-rich phase for longer, where it separates polymer domains and enlarges the openings between them, an effect reported for higher-molecular-weight PEG in PES/PEG casting systems [7]. Reducing PVP K-30 from 6 to 1 wt% removed most of the high-viscosity, chain-entangled additive that favors delayed demixing and a continuous, perforated surface in the earlier 260710-1 layers (Figure 4a); the opposing effects of PVP and PEG on PES pore structure have been reported repeatedly for cast PES membranes [7,44]. The subsequent 60 °C bath accelerated solvent exchange and fixed the open structure before it could densify, consistent with the 3.53-fold water-flow increase between the 30 and 60 °C layers. Because the three additives were changed together, these roles are a mechanistic interpretation supported by the literature rather than a factorial result; a single-variable study of the PEG 400/PEG 2000 ratio, of which dope 260825-2 (15/6 wt%) is a first instance, is identified as future work.

The matched-temperature pair isolates a useful process contrast within this revised dope. Layer 260728-3-5, coagulated at 30 °C, was 45 µm thick and passed 888 g min^−1^ of water. At 60 °C, layer 260728-3-6 was 40 µm thick and passed 3132 g min^−1^, equivalent to 147,000 L m^−2^ h^−1^. The 3.53-fold water-flow increase identified the 60 °C layer as a promising low-resistance upstream component. Its high isolated-layer flux also addressed the dilute-feed design requirement by limiting the hydraulic penalty expected from adding a second layer.

The revised surfaces display an open particulate morphology (Figure 4c,d). A later specimen from the same formulation family, 260811-1-1, shows a comparable surface organization and a porous cross-section (Figure 4e,f). The ASY base, 6H04-3, provides a contrasting, thicker component with a finer surface texture (Figure 4g,h). These images support the distinction between a thin macroporous upstream region and the manufactured base. The SEM base specimen is separate from the 6G01-4/-6 performance specimens, and the later upstream cross-section is not reassigned to the selected July coupon.

The quantitative analysis (Table 7; Figure 5) puts numbers to these images. The early 260710-1-1 surface has a projected open area of only 15%, made up of discrete openings (median D_eq 0.66 µm, D90 2.1 µm, maximum 3.6 µm), which is the perforated-skin structure described above. The revised layers have a projected open area of 41–44%, nearly three times higher, because the openings between the polymer nodules are interconnected: single connected void regions of up to 5–9 µm equivalent diameter appear in every revised field, and the area-weighted mean opening (3.9–6.8 µm) is far larger than the number-weighted median. Within the matched-temperature pair, the 60 °C layer 260728-3-6 has the same porosity as the 30 °C layer (41% vs. 42%) but coarser openings (median 0.92 vs. 0.47 µm; D90 4.2 vs. 2.0 µm), which is consistent with its 3.5-fold higher isolated-layer water flow (3132 vs. 888 g min^−1^): at equal open area, water flow scales with the size of the openings, and the 60 °C bath fixed a coarser network. The ASY base surface (6H04-3) is finer and more numerous (38% open area, median 0.24 µm, D90 0.97 µm, 366 objects in the same field), so the upstream layer is roughly four times coarser than the base at its median and more than four times coarser at D90, which is the intended prefilter ordering. The commercial references lie between the two: 23% (Sartolab 500, median 0.87 µm) and 28% (Steritop, median 1.04 µm) open area, with openings mostly below 3–5 µm. These values are projected surface openings from single fields and are compared as such; they do not measure the internal pore network or the 0.2 µm retentive structure of the base, which is characterized by bubble point and bacterial challenge (Section 2.5 and Section 3.9).

The commercial images provide additional morphological context. Sartolab 500 has rounded or elongated openings within a relatively continuous surface above a sponge-like bulk; Steritop presents a more open strut-like surface arrangement (Figure 4i–l). Different fields and scale bars preclude ranking pore sizes from the displayed panel widths. These observations motivate attention to the feed-facing morphology, while the location of broth deposits within each membrane remains an interpretation rather than an SEM result.

### 3.6. The PES Composite Overcomes the Single-Layer Performance Penalty

The selected upstream layer changed the outcome of assembly. In the explicit 31 July comparison, composite 6G01-4 + 260728-3-6 filtered 100 mL of 50-fold broth in 52.35 s, while the bare 6G01-6 reference required 185 s on the same in-house bottle-top configuration (Table 6; Figure 6a). Average flux increased from 275 to 971 L m^−2^ h^−1^, a 3.53-fold improvement and a 71.7% reduction in elapsed time. The base identifiers differ, but the manufacturing records connect them to the same dope and nominal process.

The composite was approximately 194 µm thick compared with approximately 150 µm for the base reference. Its improved broth response therefore accompanied an increase in total thickness. Together with the earlier unsuccessful stacks, this result identifies the important change as the developed upstream structure and its assembly, rather than the mere presence of a second membrane or a reduction in total film thickness.

This experiment transfers the benefit first observed with NC-assisted assemblies into the PES composite family. It preserves the existing sPES-containing asymmetric base while adding a thin, independently cast upstream region. The increase in broth flux under a common feed demonstrates the practical result sought from the composite design, without requiring a complete chemical or mass characterization of every broth constituent.

### 3.7. Interlayer Contact Is a Functional Assembly Variable

Attempts to join the membranes during phase separation showed the difficulty of maintaining contact through subsequent processing. Glycerol pretreatment reduced separation during NIPS in the recorded trial, but the membranes separated after drying. The DMSO/water pretreatments were followed by separation upon immersion. These outcomes motivated assembly after the individual layers were complete, followed by wet consolidation.

The September comparisons quantify the importance of the resulting assembly condition (Table 8). For the PES-only family, the wet-consolidated composite required 57.31 s while the loose stack required 153 s, corresponding to approximately 4910 and 1840 L m^−2^ h^−1^. For the sPES-H family, the consolidated entries required 25.45 and 32.33 s, while the loose entry required 40.04 s. Lift-off of the upper layer was observed during the filtration of unattached samples. The same practical trend therefore occurred in both formulation families.

Wrinkling, local separation and incomplete wetting can change the area participating in flow and the distribution of liquid entering the base. Interlayer air may also distinguish a dry loose stack from a wet-consolidated pair. These mechanisms were not separated experimentally, but they explain why a highly permeable upstream layer must be evaluated after assembly. The measured result supports control of the complete contact condition rather than a claim of permanent bonding or an isolated adhesion effect.

The mechanical stability of the wet-consolidated interface was assessed only within the filtration tests reported here. Under the vacuum driving force used (about 1 bar), the consolidated pairs remained in contact throughout the 100 mL charge, whereas lift-off of the upper layer was recorded only for the loose stacks. No adhesion, peel or pressure-cycling test was performed, and no long-term or pressurized operation was evaluated; wet consolidation is therefore an assembly step that establishes conformal wet contact, not a bonded laminate. In the intended normal-flow device format, the transmembrane pressure presses the upstream layer onto the base and the base onto the support screen of the housing, so the operating force acts to maintain rather than separate the interface, and delamination during filtration is not expected to be the limiting failure mode. Stability under pressure cycling, autoclaving and pressures above 1 bar, and a permanently joined (co-cast or thermally consolidated) version of the interface, remain to be established and are identified as necessary steps before device qualification.

### 3.8. Upstream sPES-H Refinement Improves the Common-Feed Comparison

The final refinement introduced sPES-H into the upstream region while retaining the sPES-containing base. Dope 0831-2 contained 7.5 wt% PES and 0.5 wt% sPES-H, corresponding to 6.25 wt% sPES-H in the total membrane-forming polymer fraction. The laboratory record described improved dissolution and a viscosity increase to 301 cP, compared with 292 cP for the PES-only reproduction dope. The isolated-layer water response decreased: 1831–2189 g min^−1^ for the sPES-H family versus 2672 g min^−1^ for the PES-only family, an approximately 18–31% reduction (Figure 7a).

Despite that lower water flow, the wet-consolidated sPES-H composites filtered broth faster. In the 2 September common-holder comparison, specimens 0831-2-3 and 0831-2-2 required 25.45 and 32.33 s for 100 mL. Their average broth fluxes were 11,100 and 8700 L m^−2^ h^−1^, compared with 4910 L m^−2^ h^−1^ for PES-only specimen 0825-1-3 (57.31 s). The individual improvements were 2.25-fold and 1.77-fold (Figure 6b and Figure 7b). Both sPES-H specimens were faster than the two commercial references in that session: Steritop required 44.42 s and Sartolab 500 required 208 s. The best composite consequently gave 1.75 and 8.17 times their respective average fluxes.

These comparisons locate the achieved performance relative to commercial membranes using one broth lot, one holder and one effective area. They also show why the upstream formulation could not be selected from clean-water flux alone. The preferred broth formulation had lower isolated-layer water flow yet a higher composite broth flux. The same feed-dependent design principle had already appeared in the base-selection comparison.

The formulation effect is reported at the specimen and assembly level. The sPES-H layers were approximately 48–50 µm thick versus approximately 40 µm for the PES-only family, and the 7.5 wt% PES control without sPES did not have a reported broth endpoint. The observations therefore support the tested sPES-H formulation, without isolating sulfonation from all resulting structural changes. Prior PES/sPES work provides a basis for considering changes in wetting and foulant interaction [31,32], and PES surface-modification studies demonstrate that protein-containing feeds can respond strongly to surface chemistry [38]. A particular electrostatic or adsorption mechanism is not required to establish the measured advantage of the present formulation.

Several physicochemical interactions could account for the higher broth flux of the sPES-H layer despite its lower water flux. First, the sulfonic acid groups of sPES-H are ionized at the near-neutral pH of the diluted broth, giving the pore walls a negative surface charge. *E. coli* cells and most cell-derived debris and proteins in a fermentation broth also carry a net negative charge at this pH, so electrostatic repulsion would reduce their adhesion to the pore walls and favor the accumulation of retained material at the open upstream surface as a loose, permeable deposit rather than inside the pores as adsorbed foulant. Second, sulfonation increases the hydrophilicity of the polymer, producing a more strongly bound hydration layer that hinders hydrophobic adsorption of proteins and lipids and improves wetting of the macroporous layer during wet consolidation; better wetting also reduces the amount of air entrapped at the interface. Third, the sPES-H layers were thicker (48–50 µm versus about 40 µm) and had a lower isolated-layer water flux, which indicates a somewhat finer or more tortuous pore network; such a layer distributes the incoming broth over more internal surface and can delay the transition from pore blockage to cake formation at the base [16,45]. The first two mechanisms are consistent with the antifouling behavior reported for PES blends with sulfonated polyarylethers [31,32,35,46] and for negatively charged or zwitterionic PES microfiltration surfaces [47] and with the sensitivity of PES microfiltration to surface chemistry in protein-containing feeds [38,39]; the September broth had been ion-exchange-processed by the supplier, and a lower ionic strength would be expected to strengthen electrostatic effects. Distinguishing among these mechanisms will require zeta-potential and contact-angle measurements of the layers and filtration with feeds of controlled ionic strength; these measurements were not part of the present development sequence, and the mechanisms are proposed as testable interpretations of the measured advantage.

### 3.9. Bacterial Retention and Protein Adsorption of the Base Grade

Because the upstream layers are macroporous, the retention of the composite is set by the 0.2 µm ASY base, and the retention of this grade was verified by bacterial challenge testing (Section 2.5; Table 9). Three pilot ASY membranes of the base family (12–12.5 wt% PES; 0, 0.5 and 0.7 wt% sPES) gave no detectable *B. diminuta* in 200 mL of permeate at challenge levels of 2.8 × 10^8^ to 1.1 × 10^9^ CFU cm^−2^ (8.4–9.0 log cm^−2^), the same outcome as the Millipore Express PLUS reference (5.8 × 10^8^ CFU cm^−2^, 8.7 log cm^−2^, no passage). Membranes with 1 wt% or more sPES showed passage (2 and 9 CFU mL^−1^ for the S25 grade at 1 and 1.5 wt%; too many to count for the S50 grade at 1 wt%), which bounds the sPES content that the base can carry without loss of integrity and is consistent with the 0.5 wt% used in the base of this study. A coupon of base 6G01-6—cast from the same dope and nominal process as the 6G01-4 base used in the composite tests (Section 2.2)—tested in September 2026 gave 42 CFU mL^−1^ in the permeate at a challenge of 3.6 × 10^8^ CFU cm^−2^ (8.6 log cm^−2^), corresponding to a log reduction of about 5.8 on a concentration basis, while the reference in the same series again showed no passage; this result is reported as measured and indicates that coupon-level integrity of the pilot base still requires control.

Water permeabilities of the tested base coupons (10,100–12,800 L m^−2^ h^−1^ bar^−1^, external measurement at 13.8 cm^2^) bracketed that of the reference (11,400 L m^−2^ h^−1^ bar^−1^), so the base grade does not owe its retention to a lower permeability. BSA adsorption on the base membranes with 0.5 and 0.7 wt% sPES (10.2 and 8.7 µg cm^−2^) and on the PES-only membrane (5.5 µg cm^−2^) was lower than on the reference (18.1 µg cm^−2^), whereas 1 wt% of the S50 grade raised adsorption to 22.6 µg cm^−2^; the lower adsorption of the sub-1 wt% sPES membranes is consistent with the hydrophilic-surface mechanism proposed in Section 3.8. Taken together, these results show that the base grade retains *B. diminuta* at sterilizing-grade challenge levels—completely in the three base-family membranes below 1 wt% sPES, and with a log reduction of about 5.8 in the composite-base coupon—so the higher broth flux of the composites is not attributable to a coarser retentive layer, although the coupon-level result shows that the base does not yet match the reference in every test. The assembled composites were not challenge-tested; because wet consolidation adds a macroporous layer upstream of an unchanged base, their retention is expected to equal that of the base, and this expectation is identified as a point to be verified in the next-generation study.

### 3.10. Interpretation of the Hypothesis and the Third-Generation Contribution

The experiments support the architectural hypothesis through a connected development sequence. The early single-sheet tests established rapid lower-concentration filtration and a performance gap under a stronger broth challenge. The ASY + NC proof-of-concept comparisons then demonstrated improvements at both 100-fold and 50-fold dilution. Developing the PES upstream layer transferred this principle to the intended polymer family, and its common-feed comparison showed a substantial improvement over the ASY base. The subsequent sPES-H and contact comparisons further improved the assembled membrane and placed its performance above the measured commercial references in the September test.

The physical interpretation is that the upstream region changes the evolution of resistance during broth filtration while adding sufficiently little hydraulic resistance of its own. A porous layer can redistribute entry flow, modify where deposits accumulate or delay the concentration of fouling at the finer region of the base. The early unsuccessful PES stacks illustrate the opposite case, in which surface resistance or poor assembly overwhelms any benefit. This interpretation is compatible with pore-blockage/deposit models [16] and with the performance benefits of appropriately selected prefilter structures in other complex feeds [24]. The present SEM and endpoint measurements do not distinguish among these possible deposition mechanisms.

The evidence is most informative when the architectural and formulation questions are kept at their demonstrated levels. Both broth dilutions were tested in the initial layered proof of concept; the final PES and PES/sPES-H specimen comparisons reported here were performed at 50-fold dilution. Accordingly, the results support the original design strategy across the development sequence and establish the final composite’s advantage in the tested broth comparisons. They do not assign an unmeasured concentration threshold or a continuous concentration-response curve to the final formulation.

Two limitations follow from this design and are stated explicitly. First, the composite endpoints are single measurements or, for the sPES-H family, two coupons from one dope prepared from a single sPES-H synthesis batch; no independent casting batches were replicated within a session, and no inferential statistics are attached to the differences. The comparisons were instead made robust in a different way: every ranking reported here was obtained with the membranes filtered on the same day, from the same prepared broth, on the same holder and at the same driving force, so that the broth, its storage history and the device are held constant within each block, and only the membrane differs. The same direction of effect was observed across independent sessions that used different broth lots and different base samples (the July composite versus base, the September PES-only and sPES-H families, and the earlier NC assemblies at two dilutions), which is the form of reproducibility available at this stage. Second, the fermentation broth was characterized only by its strain, supplier and dilution factor; cell concentration, cell-size distribution and viability were not measured, and they are known to differ between fermentation batches and with storage. This is why absolute fluxes are not compared across blocks and why the concentration conditions are described as relative dilutions rather than as solid loads. Batch-replicated casting with mean and standard deviation, and broth characterization (optical density, dry cell weight and viability) alongside each filtration block, are planned for the next-generation study, in which the multilayer hypothesis will be tested at lower dilution.

The comparison framework is deliberately application-based. A fully resolved broth solids composition is unnecessary for determining which membrane filters the same prepared feed more rapidly under the same test conditions. Lot and device are retained so that these relative results remain interpretable. The fixed-volume endpoints demonstrate improved filtration rate over the specified charge; long-run capacity and product recovery are separate performance attributes, and microbial retention has been established for the base grade rather than for the assembled composite (Section 3.9). The contribution is a reproducible fabrication concept and a documented improvement in broth filterability, developed from a pilot-manufactured asymmetric platform.

The dilution protocol also bounds the conclusions and defines the next stage of the development hypothesis. The 100 mL charges correspond to volume loads of 14.1 L m^−2^ (bottle-top) and 78.1 L m^−2^ (47 mm holder) of diluted feed, i.e., 0.28 and 1.6 L m^−2^ of undiluted broth at 50-fold dilution. The results therefore demonstrate the relative response of the membranes to broth solids delivered by normal flow, and they do not show that the present two-layer composite can process undiluted high-cell-density broth; that was not expected of it. What the results do show is that a single, separately designed upstream layer raised broth flux 3.5-fold relative to the base in the July comparison, and that the sPES-H composites exceeded both commercial references in the September comparison, whereas a single asymmetric sheet had fallen short of one reference in the February 50-fold screening. This is the outcome required to justify the second stage of the hypothesis: a fourth-generation membrane in which the upstream region is itself graded into two or more layers of decreasing openness, so that cell mass, debris and finer material are retained at successive depths before the 0.2 µm base; network models of pore-size-graded filters [48] and recent imaging of where blockage initiates in layered membranes [49] indicate that the sequence of pore sizes and the interfaces between layers govern both capacity and the initial flux decline. The next generation of the architecture will be evaluated at progressively lower dilution factors and by constant-pressure capacity (Vmax-type) testing [28], with tangential-flow filtration and depth-filter trains [13,14,25] as the process-level benchmarks. The present paper is deliberately limited to establishing the architectural principle and its measured effect at an early stage.

## 4. Conclusions

A third-generation PES composite membrane was developed by combining an sPES-containing asymmetric base with an independently tailored macroporous upstream layer. The original hypothesis—that a layered architecture could combine rapid dilute-broth filtration with an improved response to a stronger broth challenge—was supported by the development sequence. Initial ASY + NC assemblies increased average flux 4.81-fold at 100-fold dilution and 3.74-fold at 50-fold dilution relative to their corresponding bare-base controls.

The concept was then implemented as a PES composite through coordinated changes in formulation, VIPS/NIPS and layer contact. A 40 µm upstream PES layer with an open particulate morphology reduced the 100 mL endpoint on 50-fold broth from 185 to 52.35 s relative to the same-session ASY-base reference. Subsequent upstream sPES-H incorporation increased composite broth flux a further 1.77–2.25-fold relative to the tested PES-only formulation, despite lower isolated-layer water flux. Both consolidated sPES-H specimens exceeded the two commercial references in the common-holder comparison.

These results establish the practical value of the composite architecture and identify upstream morphology, formulation and wet contact as complementary design variables. They extend the SY-to-ASY manufacturing progression into a broth-oriented membrane platform whose development is guided by measured performance on the intended feed. They constitute the first stage of a staged hypothesis: that high-cell-density fermentation broths will require a multilayer membrane whose layers are individually designed, and that such a membrane can remove a large fraction of cells and debris in a single normal-flow pass. The present composite demonstrates the architectural effect under a deliberately reduced fouling load; a next-generation membrane with a graded multilayer upstream region will be developed to test the hypothesis at lower dilution and on undiluted broth.

## Figures and Tables

**Figure 1:**
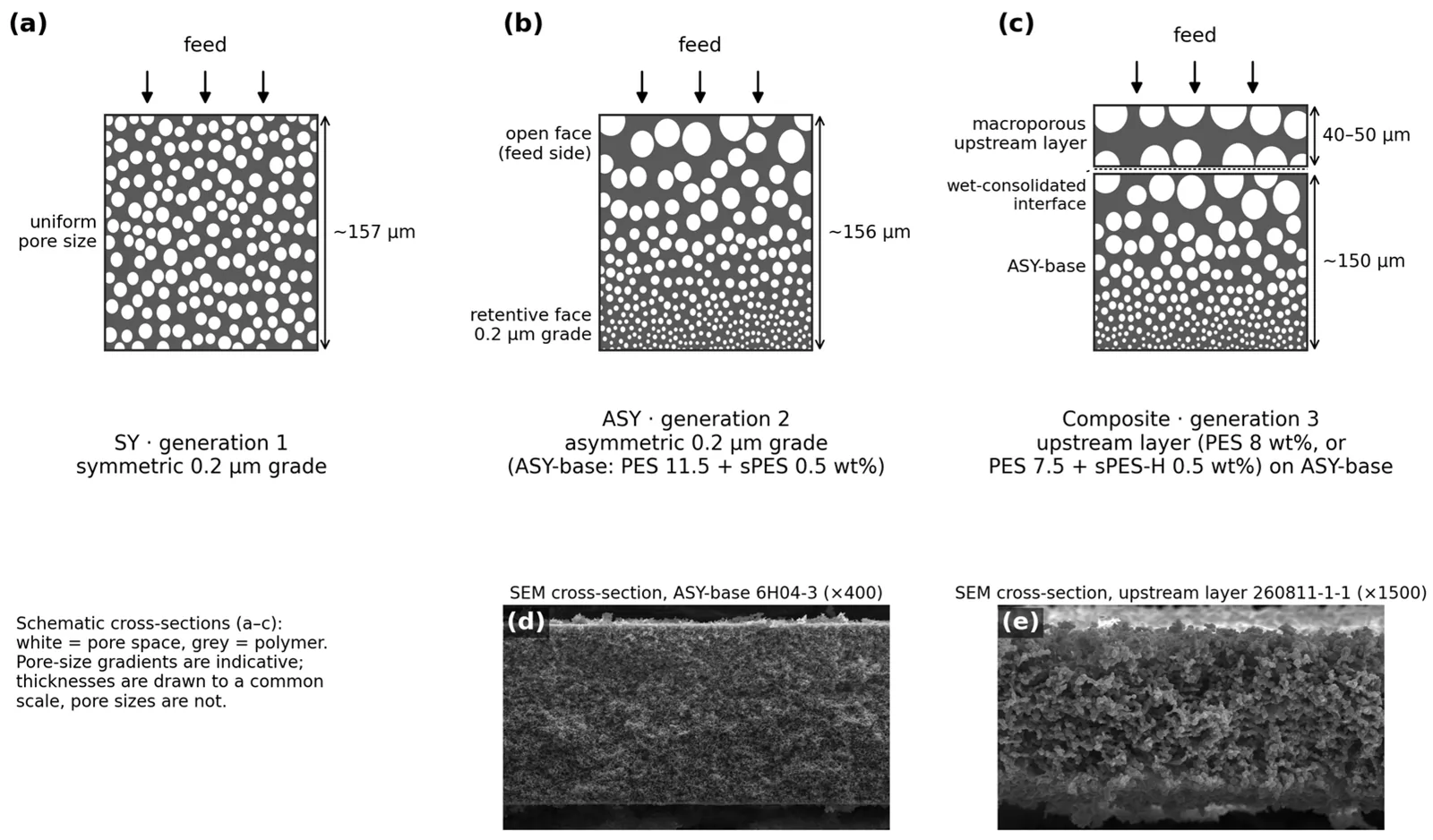
Development from SY to ASY and the third-generation composite: (**a**) symmetric SY membrane (generation 1); (**b**) asymmetric ASY membrane (generation 2), oriented with its larger-pore face toward the feed; (**c**) third-generation composite, in which the separately cast macroporous PES layer is placed upstream and wet-consolidated onto the base. The schematic cross-sections (**a**–**c**) show pore space in white and polymer in grey; pore-size gradients are indicative—layer thicknesses are drawn to a common scale and pore sizes are not. (**d**,**e**) SEM cross-sections of the ASY base (6H04-3, ×400) and of an upstream layer (260811-1-1, ×1500).

**Figure 2:**
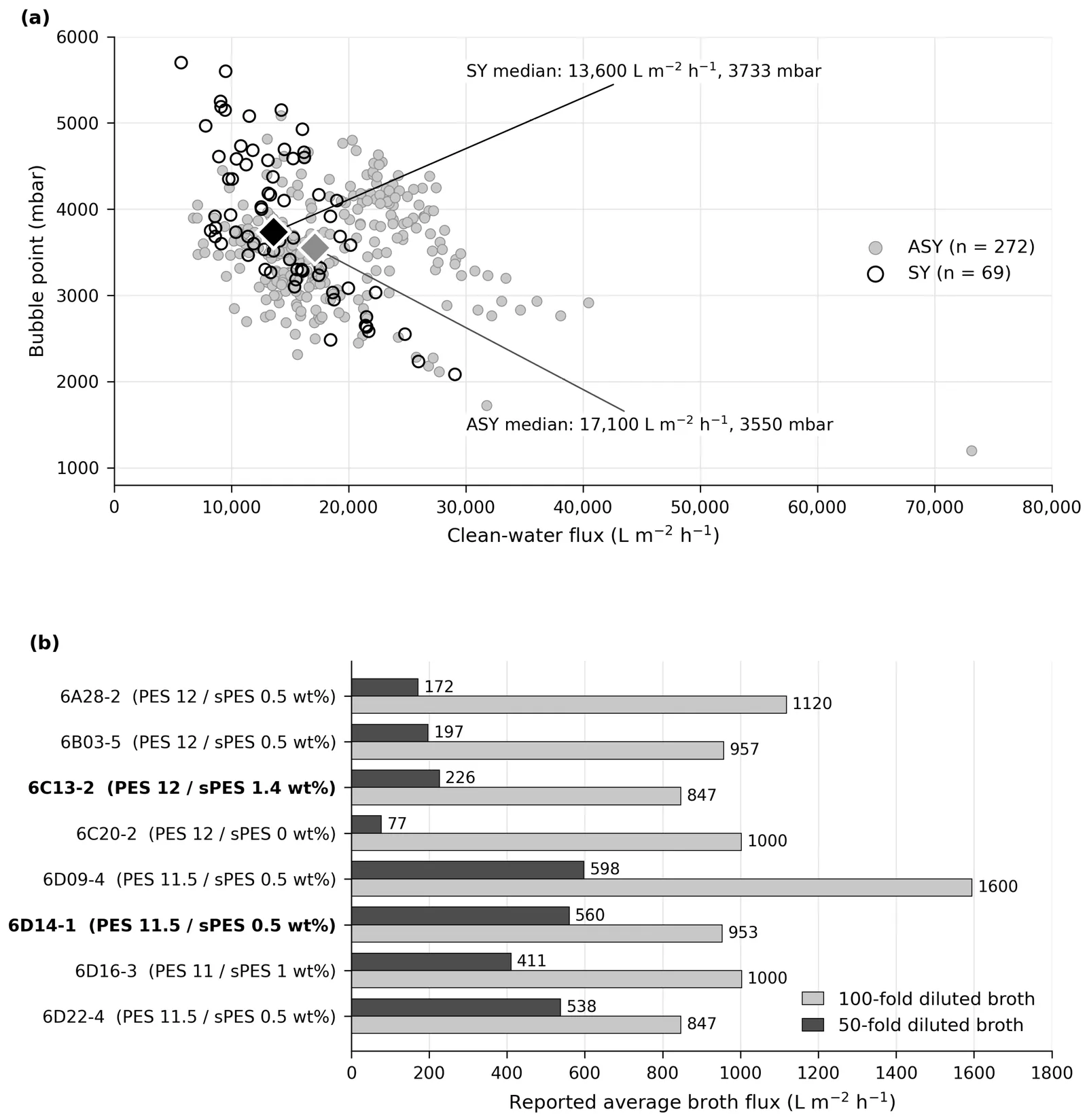
Manufacturing platform and feed-dependent base selection. (**a**) Bubble point versus clean-water flux for the joint-measurement subsets of the historical SY (*n* = 69) and ASY (*n* = 272) cohorts; diamonds mark cohort medians. (**b**) Reported average broth flux of the eight April 2026 base-development samples at 50-fold and 100-fold dilution, with PES/sPES content; 6C13-2 and 6D14-1 (bold) share a bubble point of 3650 mbar. The two panels represent separate datasets and are not pooled.

**Figure 3:**
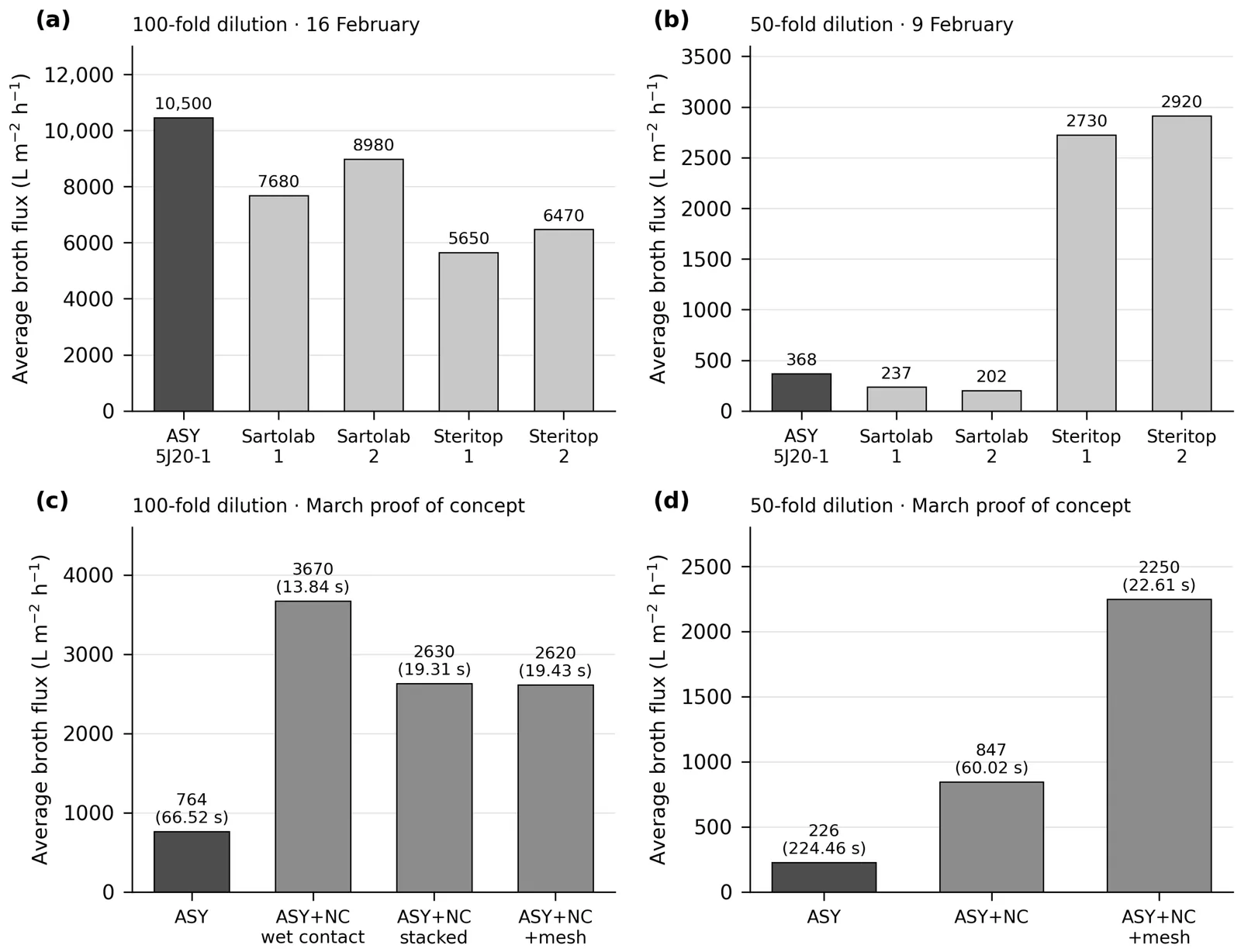
Dilution-dependent reference screening and architectural proof of concept. (**a**,**b**) ASY 5J20-1 and commercial references in the separate February 100-fold and 50-fold blocks. (**c**,**d**) March ASY + NC comparisons at 100-fold and 50-fold dilution. All endpoints are for 100 mL; areas are listed in Table 4. NC assemblies are the preliminary layered designs, not the final PES composite. Bars represent individual recorded entries, with no inferred replicate uncertainty.

**Figure 4:**
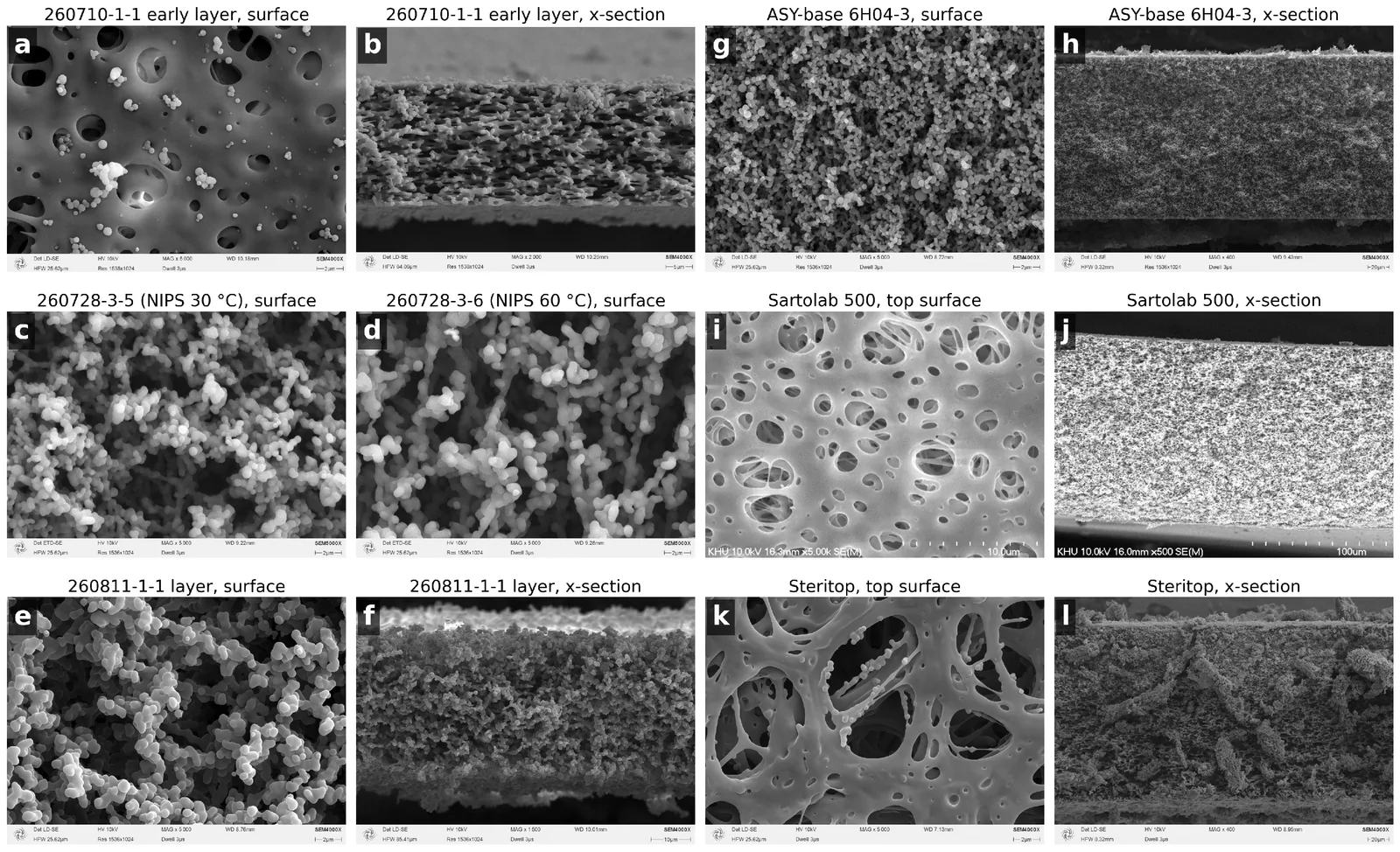
Upstream-layer, base and commercial-reference morphology. (**a**,**b**) Early 260710-1-1 surface and cross-section; (**c**,**d**) revised 260728-3-5 (30 °C NIPS) and 260728-3-6 (60 °C NIPS) surfaces; (**e**,**f**) later 260811-1-1 surface and cross-section. Original scale bars: (**a**,**c**–**e**) 2 µm; (**b**) 5 µm; (**f**) 10 µm. The original SEM fields, panel labels and instrument metadata are preserved. The later cross-section is a separate specimen from the selected July layer. (**g**,**h**) ASY base 6H04-3 surface and cross-section; (**i**,**j**) Sartolab 500; (**k**,**l**) Steritop. Original scale bars: (**g**,**k**) 2 µm; (**h**,**l**) 20 µm; (**i**) 10 µm; (**j**) 100 µm. Commercial panels have different fields and scales. Base 6H04-3 is a morphological specimen distinct from the 6G01-4/-6 performance bases. These images show separate components and do not show the assembled interface.

**Figure 5:**
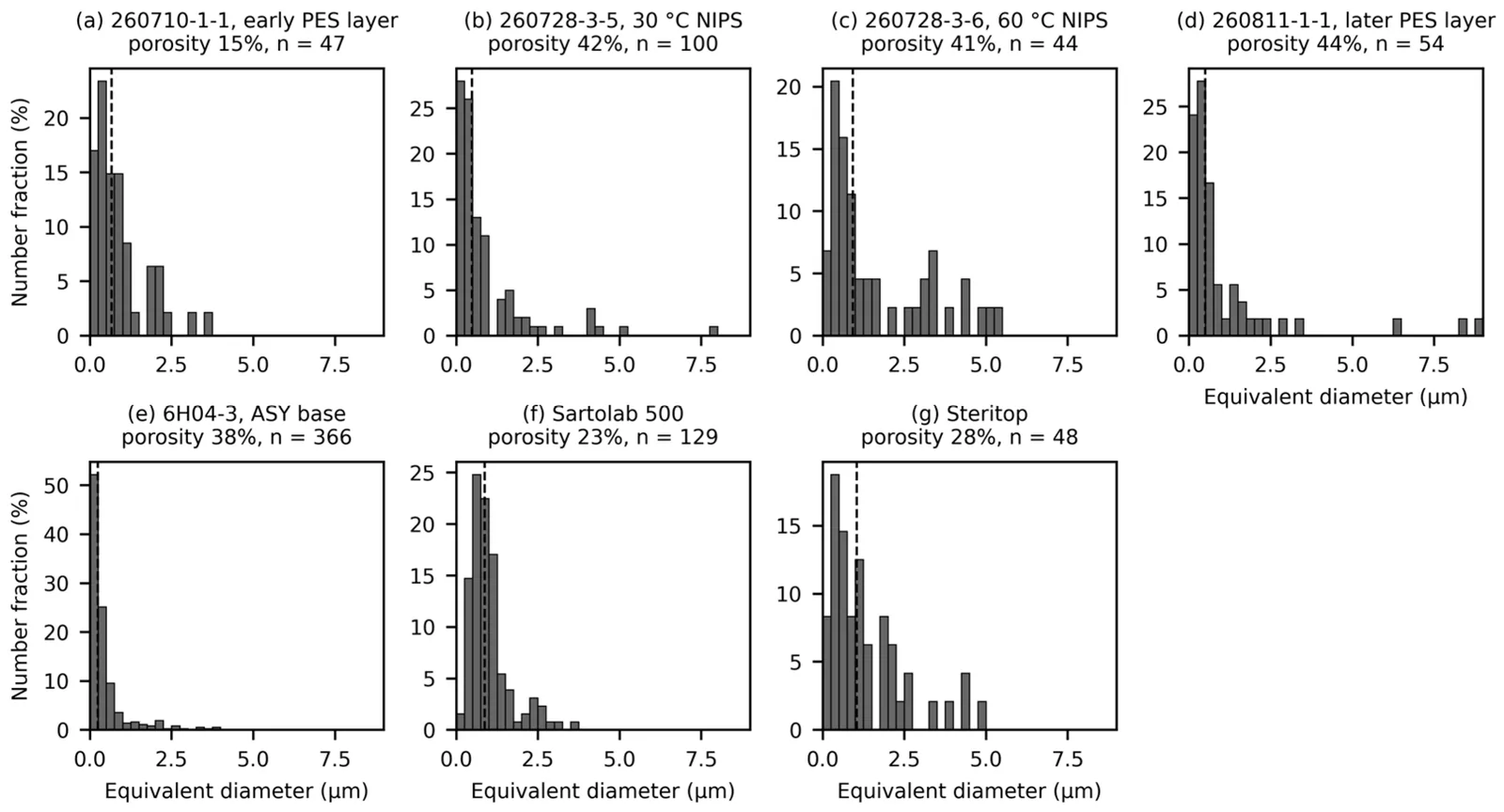
Number-weighted distributions of the equivalent diameter of surface pore openings from the ×5000 SEM fields of Figure 4: (**a**) early PES layer 260710-1-1; (**b**,**c**) revised PES layers 260728-3-5 (30 °C NIPS) and 260728-3-6 (60 °C NIPS); (**d**) later PES layer 260811-1-1; (**e**) ASY base 6H04-3; (**f**) Sartolab 500 and (**g**) Steritop references. Bin width 0.25 µm; dashed lines mark medians; the projected surface porosity and the number of objects in the size statistics are given in each panel. Summary values are listed in Table 7.

**Figure 6:**
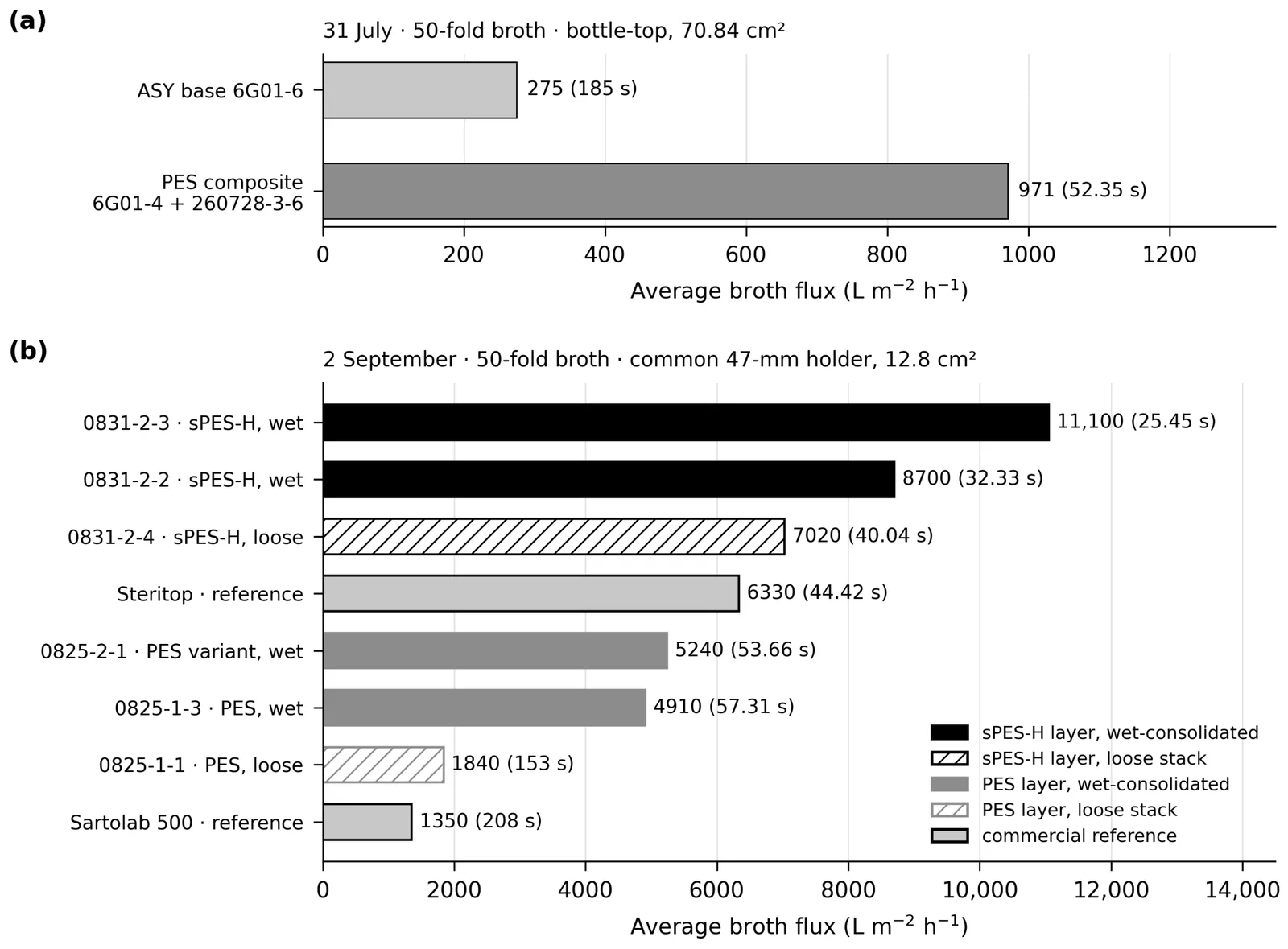
PES-composite performance in separate experimental sessions. (**a**) ASY-base reference and selected PES composite on 31 July, using the in-house bottle-top area. (**b**) The common-holder comparison on 2 September, including PES and PES/sPES-H composites and the commercial references. Numbers show average flux followed by the 100 mL time in parentheses. Hatched bars indicate loose stacks; solid bars indicate wet consolidation. Sessions use separate axes because broth and device differ.

**Figure 7:**
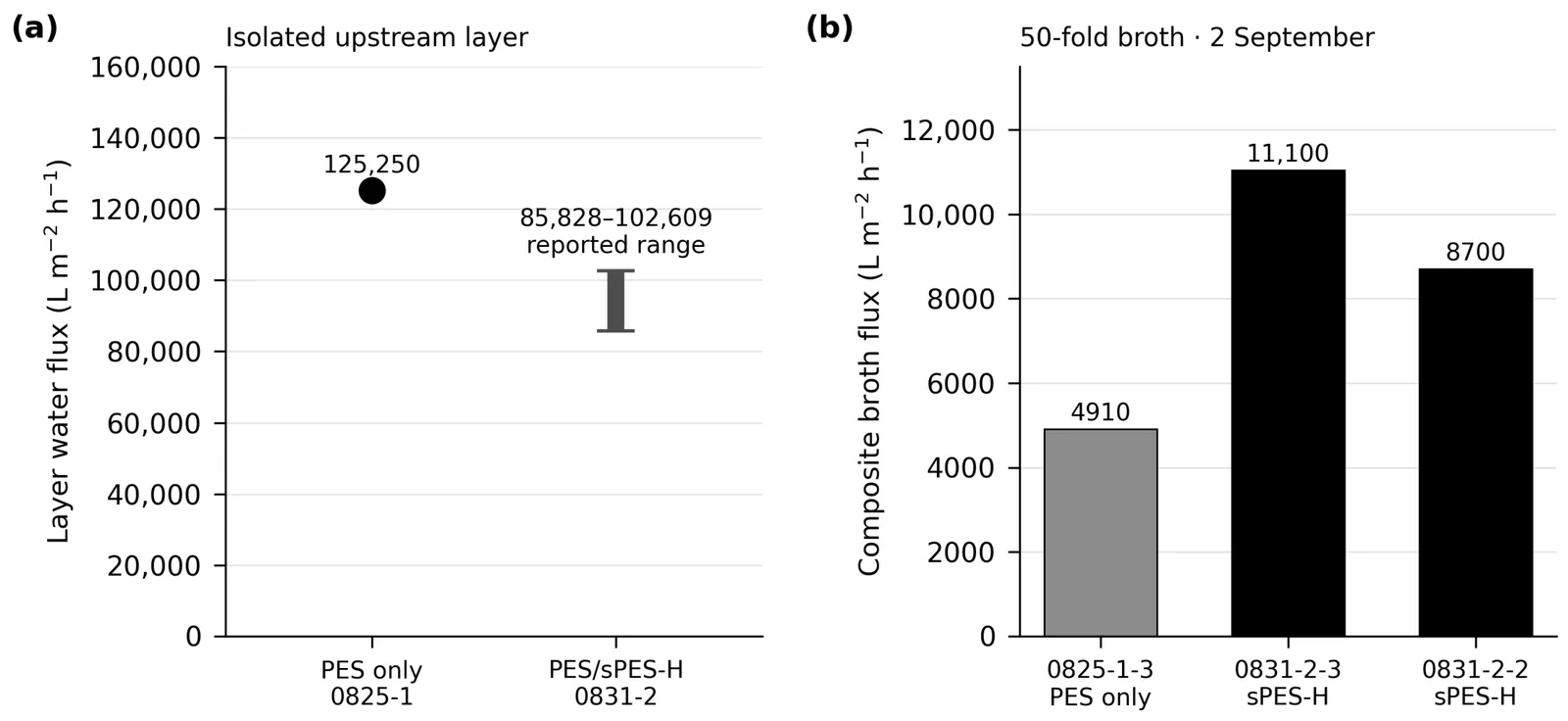
Upstream sPES-H refinement. (**a**) Isolated-layer water flux for the PES-only family and the reported range for the sPES-H family; the vertical segment is a specimen range, not an error bar or confidence interval. (**b**) Individual wet-consolidated composite endpoints expressed as average broth flux on 2 September. The two measurements concern different material states—isolated layer and assembled composite—and the water range is not assigned to either individual broth-test coupon.

**Table 1:** Principal casting-dope compositions (wt% of complete dope).

Component	ASY Base 260625-1	Early Layer 260710-1	PES Layer 260728-3	sPES-H Layer 260831-2
PES	11.5	8.0	8.0	7.5
sPES	0.5	0	0	0.5 (H-form)
PVP K-30	0.5	6.0	1.0	1.0
PEG 200	30.0	0	0	0
PEG 400	0	16.0	20.0	20.0
PEG 2000	0	0	5.0	5.0
CaCl_2_	1.0	0	0	0
LiCl	0	0.5	0.5	0.5
Ethanol	5.0	0	0	0
Butanol	5.0	0	0	0
DMSO	46.5	69.5	65.5	65.5
Total	100	100	100	100
Recorded viscosity, cP	1146	738.8	276	301

The base formulation is retained in the canonical release as A-L079. Upstream dope 260831-2 replaces 0.5 wt% PES with acid-form sPES; the sPES counterion of the base is not assigned. PP is the removable upstream casting carrier, not a retained component.

**Table 2:** Historical manufacturing cohorts and the ASY platform.

Metric	SY, Generation 1	ASY, Generation 2
Standard casting records	114	332
Production period	March–November 2024	September 2025–July 2026
Records with water throughput	69	305
Exact BP + throughput pairs	69	272
PES-only/sPES-containing records	73/41	96/236
Median thickness, µm	157 (*n* = 109)	156 (*n* = 303)
Median water flow, g min^−1^	290.33	365.80
Median water flux, L m^−2^ h^−1^	13,600	17,100
Median bubble point, mbar	3733	3550
Median dope viscosity, cP	2964	1620

Water and bubble-point medians use the joint-measurement subsets. Thickness medians use all numerical thickness observations. Cohorts are non-contemporaneous; the contrast is manufacturing context.

**Table 3:** Upstream-layer casting trials and isolated-layer water response.

Casting ID	Wet/Dry µm	VIPS min	NIPS °C/min	Water g min^−1^	Water Flux L m^−2^ h^−1^
260710-1-1	100/20	10	40/10	823.2	38,600
260710-1-3	150/30	10	40/10	87.6	4110
260710-1-4	150/30	10	60/10	334.8	15,700
260710-1-5	150/30	20	60/10	808	37,900
260728-3-5	250/45	15	30/20	888	41,600
260728-3-6	250/40	15	60/20	3132	147,000

All listed layers were dried at 50 °C for 20 min. Water flux was calculated from the source flow at 12.8 cm^2^. The selected temperature pair shares dope, wet casting thickness, vapor exposure, bath duration and drying conditions.

**Table 4:** Dilution-dependent screening and the initial layered proof of concept.

Block/Dilution	Membrane or Assembly	Area cm^2^	100 mL s	Average Flux L m^−2^ h^−1^
16 Feb/100×	ASY 5J20-1	70.84	4.86	10,500
16 Feb/100×	Sartolab 500, entries 1; 2	69	6.79; 5.81	7680; 8980
16 Feb/100×	Steritop, entries 1; 2	40	15.92; 13.911	5650; 6470
9 Feb/50×	ASY 5J20-1	70.84	138	368
9 Feb/50×	Sartolab 500, entries 1; 2	69	220; 258	237; 202
9 Feb/50×	Steritop, entries 1; 2	40	33.02; 30.88	2730; 2920
March/100×	ASY 6C13-2	70.84	66.52	764
March/100×	ASY + NC 5K11-5, wet contact	70.84	13.84	3670
March/100×	ASY + NC 5K11-5, stacked	70.84	19.31	2630
March/100×	ASY + NC + nominal 50 µm mesh	70.84	19.43	2620
March/50×	ASY 6C13-2	70.84	224.46	226
March/50×	ASY + NC 5K11-5	70.84	60.02	847
March/50×	ASY + NC + nominal 50 µm mesh	70.84	22.61	2250

The initial NC assemblies are distinct from the final PES composites. Flux comparisons are made within each block. The March workbook headers are 25 March (100×) and 27 March (50×); subsequent additions to the sheets are not assigned those dates.

**Table 5:** April base-development comparison.

Variable	6C13-2	6D14-1
PES/sPES, wt%	12.0/1.4	11.5/0.5
Dope viscosity, cP	2082	1373
Active humidification stages	1–2	1–4
Bubble point, mbar	3650	3650
Water flux, L m^−2^ h^−1^	18,100	18,400
50-fold broth flux, L m^−2^ h^−1^	226	560
100-fold broth flux, L m^−2^ h^−1^	847	953

Fluxes are retained from the original base-development comparison and dilution workbook. The samples differ in formulation and processing; the response is not assigned to sPES concentration alone. The 100-fold value of 6C13-2 in this table (847.0 L m^−2^ h^−1^) is a separate measurement made on the April dilution date and differs from the March block value of the same sample (764 L m^−2^ h^−1^, Table 4); both are retained as recorded.

**Table 6:** PES upstream development: 100 mL endpoints on 50-fold broth.

Block/Configuration	Total Thickness µm	Time s	Average Flux L m^−2^ h^−1^
Earlier: ASY base 6G01-4	153	153	332
Earlier: base + separately cast PES layer	223	351.2	145
Earlier: base + 260710-1, VIPS 10 min	173	347	146
Earlier: base + 260710-1, VIPS 20 min	183	>600	<85
31 July: ASY reference 6G01-6	150	185	275
31 July: 6G01-4 + 260728-3-6	194	52.35	971

In-house bottle-top area: 70.84 cm^2^. The earlier block contains a 2 July header and later casting IDs and is not assigned one date. The 31 July comparison is explicitly dated. Bases 6G01-4 and 6G01-6 share the dope and nominal process but are separate samples.

**Table 7:** Projected surface porosity and pore-opening size statistics from the ×5000 SEM fields (equivalent circular diameter, number-weighted unless stated).

Field/Specimen	Role	Open Area, %	n	Median, µm	Mean, µm	Area-wt. Mean, µm	D90, µm	Max., µm	Mean Inscribed Dia., µm
(a) 260710-1-1	early PES layer	15.2	47	0.66	0.90	2.26	2.09	3.64	0.73
(c) 260728-3-5	revised PES layer, 30 °C	42.2	100	0.47	0.92	4.42	2.00	7.78	0.51
(d) 260728-3-6	revised PES layer, 60 °C (selected)	40.6	44	0.92	1.67	3.92	4.24	5.30	0.91
(e) 260811-1-1	later PES layer	43.9	54	0.50	1.13	6.84	2.26	8.86	0.57
(g) 6H04-3	ASY base	37.6	366	0.24	0.46	2.28	0.97	3.85	0.23
(i) Sartolab 500	reference (second instrument)	22.7	129	0.87	1.00	1.84	1.74	3.51	0.75
(k) Steritop	reference	28.2	48	1.04	1.35	3.28	2.86	4.87	0.81

Panel letters refer to Figure 4. Open area is the projected pore-opening fraction of the analysis region (1536 × 1024 px, 25.6 × 17.1 µm; Sartolab field 576 × 432 px, 28.8 × 21.6 µm). n is the number of objects in the size statistics (border-touching objects excluded); D90, 90th percentile of the number distribution. Single fields per specimen; values describe the outermost surface and not the internal pore network.

**Table 8:** Common-holder comparison on 50-fold *E. coli* broth, 2 September 2026.

Specimen/Reference	Upstream Layer/Contact	Time s	Average Flux L m^−2^ h^−1^
0831-2-3	PES/sPES-H; wet consolidated	25.45	11,100
0831-2-2	PES/sPES-H; wet consolidated	32.33	8700
0831-2-4	PES/sPES-H; loose stack	40.04	7020
Steritop	Commercial reference	44.42	6330
0825-2-1	PES; PEG 400/2000 15/6; wet	53.66	5240
0825-1-3	PES-only; wet consolidated	57.31	4910
0825-1-1	PES-only; loose stack	153	1840
Sartolab 500	Commercial reference	208	1350

One broth lot, 100 mL, a common 47 mm holder and effective area 12.8 cm^2^. Fluxes are individual charge-average results. The two consolidated sPES-H specimens share a dope. The isolated-layer water data are 2672 g min^−1^ for 0825-1 and a family range of 1831–2189 g min^−1^ for 0831-2; these are not matched water measurements for each broth coupon.

**Table 9:** Bacterial challenge and protein-adsorption results for the 0.2 µm base grade (external laboratory, 13.8-cm^2^ coupons, 1 bar).

Membrane (ASY Pilot Sample)	PES/sPES wt% (Grade)	Water Permeability, L m^−2^ h^−1^ bar^−1^	Feed, CFU mL^−1^	Permeate, CFU mL^−1^	Challenge, CFU cm^−2^ (log cm^−2^)	BSA Adsorption, µg cm^−2^
6A09-5	12.5/0	11,065	7.7 × 10^7^	0	1.11 × 10^9^ (9.04)	5.5
6A28-3	12/0.5 (S50)	10,121	2.9 × 10^7^	0	4.13 × 10^8^ (8.62)	10.2
6C10-6	12/0.7 (S25)	11,117	2.0 × 10^7^	0	2.83 × 10^8^ (8.45)	8.7
6C11-3	12/1.0 (S50)	12,187	9.3 × 10^7^	TMTC	1.34 × 10^9^ (9.13)	22.6
6C09-3	12/1.0 (S25)	12,109	5.2 × 10^7^	2	7.46 × 10^8^ (8.87)	16.4
6C12-4	12/1.5 (S25)	11,687	3.2 × 10^7^	9	4.64 × 10^8^ (8.67)	10.0
6G01-6 (composite base; Sep 2026)	11.5/0.5	12,800	2.5 × 10^7^	42	3.6 × 10^8^ (8.6)	n.m.
Millipore Express PLUS (reference)	—	11,400	4.0 × 10^7^	0	5.8 × 10^8^ (8.7)	18.1

Brevundimonas diminuta in PBS (pH 7.4), 200 mL of permeate; BSA 1 g L^−1^ in PBS, 250 mL. TMTC, too many to count; n.m., not measured. Rows 1–6 were tested in April–May 2026 and row 7 in September 2026; the reference row gives the values reported with the April–May series, and the September series reported the same reference outcome (no passage).

## Data Availability

The historical SY/ASY manufacturing data are those of the companion study [41]; the de-identified datasets (analysis_0p2um_symmetric.csv and analysis_0p2um_asymmetric.csv) will be released with that article and are available to the editors and reviewers on request in the meantime. The formulations, casting conditions and specimen-level broth endpoints supporting the composite study are reported in Table 2, Table 3, Table 4, Table 5, Table 6, Table 7, Table 8 and Table 9. Original laboratory workbooks, casting records and microscopy files are maintained by UMTR Co., Ltd., and are available from the corresponding author on reasonable request. The historical data are reused as manufacturing context and are distinguished from the subsequent composite experiments.
